# Novel chemical starting points for drug discovery in leishmaniasis and Chagas disease

**DOI:** 10.1016/j.ijpddr.2019.05.002

**Published:** 2019-05-22

**Authors:** Irene Roquero, Juan Cantizani, Ignacio Cotillo, M. Pilar Manzano, Albane Kessler, J. Julio Martín, Case W. McNamara

**Affiliations:** a Diseases of the Developing World (DDW), Tres Cantos Medicines Development Campus, GSK, Tres Cantos, Spain; b Calibr at Scripps Research, La Jolla, CA, USA

**Keywords:** Visceral leishmaniasis, Chagas disease, Drug discovery, HTS, Open innovation

## Abstract

Visceral leishmaniasis (VL) and Chagas disease (CD) are caused by kinetoplastid parasites that affect millions of people worldwide and impart a heavy burden against human health. Due to the partial efficacy and toxicity-related limitations of the existing treatments, there is an urgent need to develop novel therapies with superior efficacy and safety profiles to successfully treat these diseases. Herein we report the application of whole-cell phenotypic assays to screen a set of 150,000 compounds against *Leishmania donovani*, a causative agent of VL, and *Trypanosoma cruzi*, the causative agent of CD, with the objective of finding new starting points to develop novel drugs to effectively treat and control these diseases. The screening campaign, conducted with the purpose of global open access, identified twelve novel chemotypes with low to sub-micromolar activity against *T. cruzi* and/or *L. donovani*. We disclose these hit structures and associated activity with the goal to contribute to the drug discovery community by providing unique chemical tools to probe kinetoplastid biology and as hit-to-lead candidates for drug discovery.

## Highlights

• An open source drug discovery screen between The Tres Cantos Open Lab Foundation and Calibr at Scripps Research.
• High-throughput phenotypic screen of a 150,000-compound library against *T. cruzi* and *L. donovani*.
• Identification and characterization of 12 novel chemical series.
• 7 of those 12 chemical series are active against both *T. cruzi* and *L. donovani*.
• These chemical series may be valuable tools to identify new drug targets.

## Graphical abstract

**Figure:**
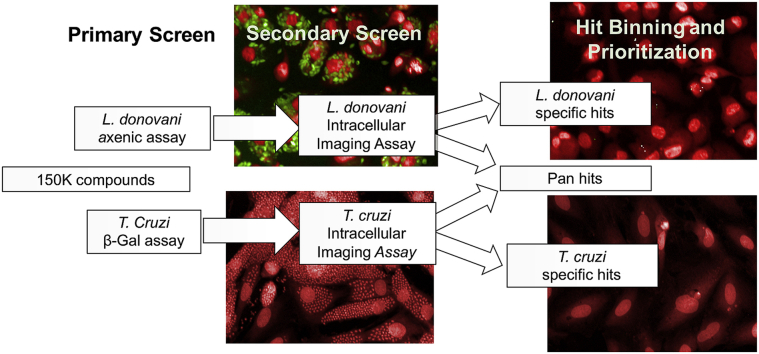

## 1 Introduction

Chagas disease (CD) and visceral leishmaniasis (VL) are both neglected tropical diseases (NTDs) with several aspects in common: they are both vector-borne diseases caused by parasites included in the kinetoplastid class, *Trypanosoma cruzi* and *Leishmania* spp., respectively; they are considered high-morbidity, low-mortality diseases; the available treatments are inadequate and new drugs are urgently needed (WHO, 2017).

This paper reports the application of high-throughput, whole-cell phenotypic assays to screen a set of 150,000 compounds from Calibr at Scripps Research against axenic *L. donovani* amastigotes and *T. cruzi* trypomastigotes with the objective of finding new starting points to develop improved drugs to treat visceral leishmaniasis and Chagas disease. Applying learnings from clinical development, *T. cruzi*-active hits were prioritized for compounds that imparted cidality against the trypomastigote and amastigote stages of the lifecycle. Whereas the L. *donovani* screen cascade progressed from hit compounds against axenic amastigotes into a counterscreen against intramacrophage amastigotes to ensure greater physiological relevance for final hit compound prioritization.

### 1.1 Visceral leishmaniasis

Based on clinical presentation, leishmaniasis is classified into three distinct syndromes: cutaneous, mucocutaneous and visceral leishmaniasis. The last one, also known as *kala-azar*, is caused by *Leishmania donovani* and *Leishmania infantum*, although clinical manifestation not only depends on the parasite species but also on the patient immune response (Georgiadou et al., 2015). It is estimated that 12 million people suffer from leishmaniasis worldwide and 350 million, across 88 countries, remain at risk of infection. Cutaneous leishmaniasis, caused by more than ten *Leishmania* species, is widely distributed with 0.7–1.2 million new cases per year, whereas 90% of VL cases are reported in just six countries: India, Bangladesh, Sudan, South Sudan, Brazil and Ethiopia. It is estimated that 0.2–0.4 million cases of VL occur every year (Alvar and Arana, 2017; Alvar et al., 2012).

*Leishmania* spp*.* have a complex life cycle with two distinct parasite stages: the promastigote form, found in the sand-fly vector, and the amastigote form, present inside several types of mammalian host cells. Briefly, the infection begins with the bite of an infected female sandfly for blood feeding. During the bite, promastigotes are released into the mammalian skin, where they invade several types of cells such as macrophages. The promastigote is then internalized into a parasitophorous vacuole where it transforms into an amastigote. After an intense amastigote replication, the macrophage membrane ruptures releasing the amastigotes into the surrounding tissue. These amastigotes can invade new cells or even be ingested by a new sandfly vector during a subsequent blood meal (Teixeira et al., 2013).

Unlike the other leishmaniasis presentations, the fatality rate of VL without treatment is approximately 90%, often due to hemorrhagic or infectious complications of the disease. The current therapeutic arsenal against VL includes pentavalent antimonials, amphotericin B, miltefosine, paromomycin and pentamidine. More recently, liposomal amphotericin B has been adopted as the front-line treatment of VL because it has the highest efficacy and lower safety risks amongst all the available drugs. However, amphotericin B can be cost-prohibitive in resource-limited settings and it requires intravenous administration. On the other hand, miltefosine is orally administered, but is contraindicated in women who are pregnant or of child-bearing potential. While the ideal treatment would have a low cost-of-goods, be administered orally, have minimal toxicity, not be contraindicated, and be highly efficacious, it is important to develop drugs with a novel mechanism of action to overcome the emerging drug-resistant parasites (Georgiadou et al., 2015; No, 2016).

### 1.2 Chagas disease

Clinically, CD has two phases of manifestation: the acute and the chronic phase. During the acute phase, which usually remains asymptomatic, parasites can be detected by blood PCR during the first 4–8 weeks following infection. The subsequent activation of the immune system leads to a decrease in the parasite load and triggers the start of the chronic phase. Without treatment, the chronic phase will persist for the remainder of the patient's life. Although 60–70% of patients with a chronic infection will never progress into symptomatic disease, the remaining 30–40% of chronically infected individuals will suffer a severe cardiac or digestive syndrome within 10–30 years after the initial infection (Chatelain, 2015). According to WHO, around 6–7 million people are infected and 75 million are at risk of infection with *T. cruzi*, mainly in Latin American countries (WHO, 2017).

During its life cycle, *T. cruzi* parasites alternate between different morphological and functional stages: the amastigote and epimastigote forms are replicative stages found in the mammalian host and in the triatomine vector, respectively, while trypomastigotes are the infective form present in both hosts. The infection begins when a triatomine insect bites the mammalian host and defecates into the skin wound. The feces of an infected triatomine have trypomastigotes, which can successfully establish infection. Once inside the mammalian host, the parasite can invade several types of nucleated cells. In the cytoplasm, trypomastigotes transform into amastigotes, which replicate intensively. Then amastigotes differentiate again into trypomastigotes and the host cell ruptures releasing those parasites into the blood. The circulating trypomastigotes can invade new cells or be ingested by a triatomine vector during a blood meal. During the vector stages of the life cycle, trypomastigotes will transform into epimastigotes, replicate and differentiate into infective metacyclic trypomastigotes that will be excreted with the feces (Bern, 2015; Teixeira et al., 2012). Thus, within the human host, both the trypomastigotes and amastigote stages should be targeted, either by a dual-acting compound or by a combination therapy, to achieve maximal efficacy.

Benznidazole and nifurtimox, the current treatment options for Chagas disease, are only effective during the acute phase of the disease and present important adverse effects (Sales Junior et al., 2017). Benznidazole can produce hypersensitivity, bone marrow suppression and peripheral neuropathy, while nifurtimox usually produces gastrointestinal maladies and up to 30% of the patients might develop central nervous system perturbations. Importantly, nifurtimox use represents a higher risk of heart failure when compared to benznidazole (Bermúdez et al., 2016). The first drugs tested in recent clinical trials were azoles such as posaconazole, based on promising preclinical data; however, posaconazole was found to be trypanostatic, which likely contributed to low clinical efficacy. In the STOP-CHAGAS trial, posaconazole and benznidazole exhibited efficacy of 19% and 62%, respectively (Morillo et al., 2017a). These findings underscore the importance of assessing cidality of compound hits at the onset of drug development to avoid wasted efforts and resources on compounds that only exert static effects on parasite development within the human host. Moreover, in the case of benznidazole-treated group, 32% of the patients had to permanently discontinue treatment due to side effects. Again highlighting the urgency to develop new treatments with greater efficacy in both acute and chronic phase that will also demonstrate improved safety profiles over the currently available drugs (Bern, 2015, 2017; Morillo et al., 2015, 2017a, 2017b).

### 1.3 Working together to tackle NTDs

A collaborative initiative between GSK and Calibr at Scripps Research (Calibr) was set through the Tres Cantos Open Lab Foundation (TCOLF) with the aim of providing new chemical starting points to tackle these diseases (Ballell et al., 2016).

High throughput screening (HTS) has become the classical drug discovery approach to evaluate large chemical libraries to identify novel lead series to address the lack of efficacious and safe treatments for these NTDs. Amongst the screening strategies, either target-based or phenotypic screens may be deployed to discover small-molecule inhibitors, and each has its advantages and limitations. In the field of anti-infective drug discovery, target-based screening shows a high attrition rate, partly due to the poor translation from biochemical to cellular activity and *in vivo* efficacy. Many factors, such as cell permeability and even non-essentiality of the target in the context of the human host, can contribute to this translational disconnect. Thus, phenotypic assays are the preferred screening strategy for anti-kinetoplastid drug discovery (Don and Ioset, 2014; Martín et al., 2018) to overcome many of those limitations created in a target-based screen. A consequence of phenotypic screening is that the corresponding drug target is unknown until target deconvolution studies can be conducted on the hit series. An important consider for phenotypic screening campaign is that the screen cascade must balance those assays that permit high-throughput screening to manage large compound libraries yet preserve key secondary assays to characterize screen hits for prioritization of clinically relevant starting points.

## 2 Materials and methods

### 2.1 Compound library and resupply

The Diversity Library from Calibr is comprised of small molecule compounds derived from commercial and propriety sources totaling ∼150,000 compounds. This library was prepared as a 2 mM stock solution dissolved in DMSO and maintained in 1536-well low dead volume source plates (plate # LP-0400; Labcyte Inc) that are compatible for acoustic transfer. Final screen concentration (5 μM) was dictated by the % DMSO tolerance of the parasites.

For screening, assay-ready plates were used because of the limitation of high-throughput compound transfer equipment in the BSL3 laboratory at GSK (Tres Cantos site). Generally, assay-ready plates were prepared at Calibr, then the sealed plates were shipped on dry ice to GSK in Tres Cantos. Plates were thawed overnight prior to cell dispense and briefly centrifuged (30 s at 100×*g*) to ensure all compound was collected at the bottom of the assay well before removal of the seal. Specifically, 1536-well assay-ready plates were prepared by acoustically transferring 30 nL of compound stock per well, and 250 nL was transferred per well for 384-well plates by using an Echo liquid handler (Labcyte, Inc.). Plates were immediately sealed and placed at −20 °C storage until shipment on dry ice. Single shot (SS) experiments were run at 5 μM final compound concentration. For dose response (DR) experiments, 11-point, one-to-three dilution series were prepared with 50 μM as starting concentration for *L. donovani* and *T. cruzi* assays and 100 μM for HepG2 assay.

For the hit reconfirmation phase, the available powder resupply stocks were purchased from the commercial vendors that sourced the screen compounds. 76 *T. cruzi* hits and 63 *L. donovani* hits were available for resupply from ChemDiv and Life Chemicals. All compound purity (>95%) and molecular mass were confirmed using liquid chromatography-mass spectrometry analysis using a Single Quad ZQ (Waters) coupled to an Extend-C18 Rapid Resolution 3.5 μm HPLC column (Waters).

### 2.2 Parasites and mammalian cell cultures

THP-1 cells (human monocytic leukemia) were made available by GSK Biological Reagents and Assay Development Department (BRAD, Stevenage, UK). LdBOB axenic amastigotes expressing eGFP were kindly provided by Manu de Rycker from Dundee University and used for all *L. donovani* assays. LLC-MK2 cells (green monkey kidney epithelial cells) were purchased from the European Cell Cultures Collection (ECACC reference 85062804) and used to expand the *T. cruzi* parasite population. NIH-3T3 cells (mouse endothelial fibroblasts) were made available by GSK-Biological Reagents and Assay Development Department (BRAD, Stevenage, UK). H9c2 (rat cardiomyocytes) were purchased at the European Cell Cultures Collection (ECACC, Salisbury, UK). *T. cruzi* parasites from the Tulahuen strain expressing β-galactosidase were kindly provided by Dr. Buckner (University of Washington, Seattle, USA) and used for all *T. cruzi* assays. Parasites were maintained in culture by weekly infection of LLC-MK2 cells. Trypomastigote forms were obtained from the supernatants of LLCMK2 infected cultures harvested between days 5 and 9 of infection, as described in 2.4.2.

### 2.3 Leishmania donovani

#### 2.3.1 Axenic amastigote assay

For the primary screen assay, 6 μL per well of eGFP LdBOB axenic amastigotes at a final concentration of 1 × 10^5^ cells/mL in amastigotes assay media containing 500 U/mL penicillin/streptomycin (Invitrogen™) were dispensed in the assay plates (Greiner #782092). Control columns (100% growth inhibition) contained 6 μL per well of amastigotes assay media. Plates were incubated for 72 h, at 37 °C, 5% CO_2_. Then 2 μL per well of a resazurin solution (0.2 μM in phosphate buffered saline (PBS) with Igepal (Sigma-Aldrich), 0.2% (v/v)) were added and plates were incubated for 4 h at room temperature before fluorescence signal was detected. Cells were counted using a CASY cell counter (Roche-Applied Science), plates were dispensed with a Multidrop Combi dispenser (Thermo Scientific) and a PerkinElmer Envision (excitation/emission at 528/590 nm) was used as plate reader. This assay was adapted from Peña et al. (2015).

#### 2.3.2 Intra-macrophage *L. donovani* (InMac) assay

For this High Content Imaging assay, THP-1 cells were counted using a CASY cell counter and were differentiated in 225 cm^2^ T- FLASK in the presence of 30 nM PMA (phorbol 12-myristate 13-acetate) (Sigma-Aldrich) at a final concentration of 6 × 10^5^ cells/mL. Following incubation for 24 h at 37 °C, 5% CO_2_, cell differentiation was visually confirmed using an optical microscope. Cells were washed using cell culture media (RPMI (Invitrogen™), 10% FBS (Gibco), 25 mM Hepes (Invitrogen™), 1.25 mM Sodium pyruvate (Invitrogen™), 2.5 mM L-Glutamine (Invitrogen™)) and then were infected using eGFP LdBOB axenic amastigotes (counted with a CASY cell counter) at a multiplicity of infection of 10 (i.e. 6 × 10^6^ parasites/mL) in the T-FLASK containing differentiated THP-1 cells. After overnight incubation, the remaining parasite was removed washing each T-FLASK with sterile PBS and the infected cells were harvested by treatment with 0.05% (w/v) trypsin plus 0.48 mM EDTA for 5 min. A cell preparation with a final concentration of 2 × 10^5^ cells/mL was prepared in assay media consisting of RPMI (Invitrogen™), 2% FBS (Gibco) and 25 mM sodium bicarbonate (Invitrogen™) containing 30 nM of PMA. 50 μL of infected cells were plated onto 384-well plates (Greiner #781091) containing compounds using a Multidrop Combi dispenser. Non-infected cells were used as positive control of 100% compound response and were counted and 50 μL were dispensed in control columns. Plates were incubated for 96 h at 37 °C, 5% CO_2_ and then fixed with a 4% (v/v) formaldehyde-PBS solution for 30 min at room temperature. Then, wells were washed with PBS and stained with 10 mg/mL DAPI plus 0.1% (v/v) Triton X-100 in PBS for 30 min at room temperature using an EL406 multi well platewasher (BioTek). After a second washing step with PBS, 50 μL of PBS were added to each well. Then, plates were read on a high-content microscope (Opera QEHS) using a 20 × objective, 3 fields per well. Two exposure images were taken for each well using 405 nm and 488 nm laser excitation. Automated image analysis was performed with a script developed on Acapella^®^ High Content Imaging and Analysis Software (PerkinElmer). Three outputs were provided for each sample well: (1) THP-1 cell count to determine drug-related cytotoxicity (aka Mac); (2) average number of amastigotes per macrophage as infection level measurement (aka AmMac); and (3) percentage of infected cells per well as a second infection marker (aka InfCel). This assay was adapted from Peña et al. (2015).

#### 2.3.3 *L. donovani* inMac horse serum assay

This assay was previously described by Tegazzini et al. (2016). In brief, the procedure is the same as for InMac assay described in 2.3.2, although assay media contains 2% horse serum (Gibco) instead of FBS. Horse serum (HS) promotes the differentiation of intracellular parasites to a more amastigote-like form and permits the evaluation of compound effect on intracellular parasite replication. Specifically, the *in vitro* replication rate more closely mimics *in vivo* rates (2-day doubling time) and there is no discernible effect on host cell viability (Tegazzini et al., 2016). Three outputs are measured for each sample well: (1) THP-1 cell count to determine drug-related cytotoxicity (aka Mac-HS); (2) average number of amastigotes per macrophage as infection level measurement (aka AmMac-HS); and (3) percentage of infected cells per well as a second infection marker (aka InfCel-HS).

### 2.4 Trypanosoma cruzi

#### 2.4.1 β -galactosidase assay

For the primary screen assay, 6 μL per well of a solution containing 1.67 × 10^5^ NIH-3T3 cells/mL and 1.67 × 10^5^ trypomastigotes/mL in DMEM assay media was dispensed in the 1536-well plates (Greiner #782092) with a Multidrop Combi dispenser. Control columns (100% growth inhibition) contained 6 μL per well of 1.67 × 10^5^ trypomastigotes/mL solution. Plates were incubated for 96 h, at 37 °C, 5% CO_2_. Then 2 μL per well of a 20 μM resorufin-β-D-galactopiranoside (Sigma-Aldrich) solution (in PBS supplemented with 0.2% Igepal) were added and plates were incubated for 4 h at room temperature before fluorescence signal was detected using a PerkinElmer Envision plate reader (excitation/emission at 531/595 nm). This assay was adapted from Peña et al. (2015).

#### 2.4.2 *T. cruzi* intracellular imaging assay

For this high-content imaging assay, H9c2 cells were seeded in 225 cm^2^ T- FLASK in DMEM with 10% FBS for 4 h to allow attachment. *T. cruzi* trypomastigotes, collected at days 5–8 after infection from LLC-MK2 parasite infected cultures, were allowed to swim out for 4 h at 37 °C from a centrifuged pellet (1600×*g*/10 min). Trypomastigotes were then collected and counted in a CASY Cell Counter. Trypomastigotes, in supplemented DMEM, were added to H9c2 cultures in a multiplicity of infection of 1 and incubated for 18 h. Cells were washed once with PBS before incubation of the infected H9c2 monolayer with trypsin (Life-Technologies) to detach cells from the flask. Cells were counted in a CASY Cell Counter and their density set at 5 × 10^4^ cells/mL in supplemented DMEM. Infected H9c2 were dispensed into 384-well plates at 50 μL per well using a Multidrop Combi dispenser. After seeding them, the plates were incubated at 37 °C, 5% CO_2_ for 72 h. Cultures were then fixed and stained with 50 μL per well of an 8% formaldehyde and 4 μM DRAQ5 DNA dye (BioStatus, UK) in PBS solution. Then plates were read in a PerkinElmer Opera QEHS using a 20 × objective, 5 fields per well. DRAQ5 signal was detected using 635 nm excitation laser and a 690/50 emission detection filter. Automated image analysis was performed with a script developed on Acapella^®^ High Content Imaging and Analysis Software (PerkinElmer). Three outputs were provided for each sample well: (1) number of host cells nuclei to determine drug-related cytotoxicity (aka H9c2); (2) number of amastigotes per cell as infection level measurement (aka AmCel); and (3) percentage of infected cells per well as a second infection marker (aka InfCel). This assay was adapted from Alonso-Padilla et al. and Peña et al. (Alonso-Padilla et al., 2015; Peña et al., 2015).

#### 2.4.3 Trypomastigote assay

The trypomastigote assay was run in 384-well plates (Greiner #781074) by dispensing 50 μL of a solution of 1 × 10^6^ trypomastigotes/mL in culture media (DMEM (Invitrogen™), 2% FBS (Biowest), 200 U/mL penicillin/streptomycin (Invitrogen™)). *T. cruzi* trypomastigotes were obtained as described in section 2.4.2. Control columns (100% growth inhibition) contained 50 μL per well of culture media. Plates were incubated for 24, 48 or 72 h at 37 °C, 5% CO_2_ and then developed using CellTiter-Glo^®^ Reagent (50 μL per well) using a Multidrop Combi dispenser. Plates were left for 10 min at room temperature for stabilization and then luminescence was read using a PerkinElmer Envision plate reader.

### 2.5 HepG2 cytotoxicity assay

This assay was previously described by Peña et al. (2015). Briefly, actively growing HepG2 cells were harvested and a cell suspension at a final density of 1.2 × 10^5^ cells/mL in Eagle's MEM (containing 10% FBS, 1% NEAA, 1% penicillin/streptomycin) was dispensed into 384-well plates (Greiner #781091) using a Multidrop Combi dispenser (25 μL, 3000 cells per well). Plates were incubated for 48 h at 37 °C, 5% CO_2_, then developed using CellTiter-Glo^®^ Reagent (25 μL per well) using a Multidrop Combi dispenser. Plates were left for 10 min at room temperature for stabilization and then read using a PerkinElmer ViewLux reader.

### 2.6 Data analysis

Data were normalized to percent of biological response by using positive (i.e. highest response achieved by a chemical tool compound, RCtrl2) or negative (i.e. lowest response achieved in the absence of any testing compound, RCtrl1) controls by using the following equation:

$$\text{\%Response}=\frac{\left|\text{Rctrl}1-\text{Rx}\right|}{\left|\text{Rctrl}1-\text{Rctrl}2\right|}\times 100$$

where Rx is the assay response measured for the compound X. RCtrl1 and RCtrl2 are calculated as the average of replicates in the same microtiter plate where the compound X is tested.

Assay performance statistics, such as signal to background ratio, Z’ and robust 3 × SD activity cutoff (Zhang, 1999) were calculated using templates in ActivityBase XE (IDBS, Guilford, Surrey, UK). Hit population analysis and visualization were conducted using Spotfire DecisionSite (Spotfire, Inc., Somerville, Massachusetts). pIC_50_ values (pIC_50_ = - log IC_50_) were obtained using the ActivityBase XE nonlinear regression function in the full curve analysis bundle. Dose response curves were fitted to the 4-parameter logistic equation curve with a floating upper asymptote in the range of 80–120%. To address toxicity, compound selectivity index (SI) was calculated as SI = pIC_50_ Antiparasitic output - pIC_50_ Mammalian cell line (THP1/H9c2/HepG2).

### 2.7 Biosafety

Several parasite and mammalian cell lines were used in the assays described above: *Leishmania donovani* LdBOB axenic amastigotes expressing GFP, *Trypanosoma cruzi* Tulahuen strain expressing β-galactosidase, THP-1 (human monocytic leukemia) cell line, LLC-MK2 (green monkey kidney epithelial cells), NIH-3T3 (mouse endothelial fibroblasts), H9c2 (rat cardiomyocytes) and HepG2 (liver hepatocellular carcinoma cells).

Experimental work with live *L. donovani* or *T. cruzi* cells were carried out following standard operating procedures in compliance with biosafety level 3 regulations. HepG2 and THP-1 cells were treated according to GSK policies for management of human biological samples.

## 3 Results and discussion

When designing an HTS campaign, it is important to choose the most relevant *in vitro* assay in the disease to improve translation to *in vivo* and clinic results because significant differences may exist in drug sensitivity between different life-cycle stages of the parasite (Martín et al., 2018). However, it is worth noting that to screen large compound libraries it is necessary to employ assays with enough throughput and cost-effectiveness to justify this approach. This consideration is paramount for biosafety level 3 (BSL3) pathogens, such as *L. donovani* and *T. cruzi*, which require stringent safety protocols, but may limit access to screening automation available to BSL1 or 2 agents. Therefore, the primary screens against *L. donovani* and *T. cruzi* in this screening campaign employed practical, high-throughput, cost-effective primary screens conducive in a BSL3 setting then counterscreened against the reduced set of screen hits in a more relevant, lower throughput assay to further downselect to higher priority compound hits. Our hypothesis was that this strategy would provide a high-throughput approach to identify starting points for kinetoplastid drug discovery. Furthermore, for *T. cruzi*, an additional assay to assess trypomastigote cidality was employed to further characterize the value of compound hits.

### 3.1 *L. donovani* HTS campaign

Measuring activity of compounds in antiparasitic *in vitro* assays requires recapitulating the disease conditions, including parasite life-cycle stage and relevant host cells. Taking this into account, *L. donovani* amastigotes have been identified as disease-relevant form of the parasite (Martín et al., 2018).

However, it is not rationale to exclusively assay axenic parasites because *L. donovani* is known to invade host cells and establish intracellular infections. Although an axenic assay has the advantage of being a high-throughput assay, it also has major drawbacks: axenic amastigotes are different from intracellular amastigotes regarding protein expression and drug susceptibility, permeability barriers and pH gradients introduced by the phagolysosome are not recapitulated in the assay media, and compounds affecting parasite-host interaction cannot be identified with this assay (De Rycker et al., 2013). Necessitating a balance between assay throughput in a BSL3 environment and physiological relevance, hits selected with the less physiologically relevant axenic hit compounds were re-evaluated against a representative intramacrophage (InMac) assay, that provides a more disease-relevant screening condition. The InMac assay is a high-content imaging assay which is allows for concomitant measurement of multiple parameters to inform on the effects of the screen compound against both the parasite and host cell. The multi-parametric readout is powerful for hit analysis and includes measurement of the number of amastigotes per macrophage (AmMac), the percentage of infected cells (InfCel) to measure compound efficacy, and measurement of compound cytotoxicity against the human macrophage THP-1 host cells (Mac output). Thus, non-toxic compounds showing more potent half maximal inhibitory concentration (IC_50_) in the efficacy outputs, AmMac and InfCel, can be prioritized for demonstrating selective activity.

**Fig. 1:**
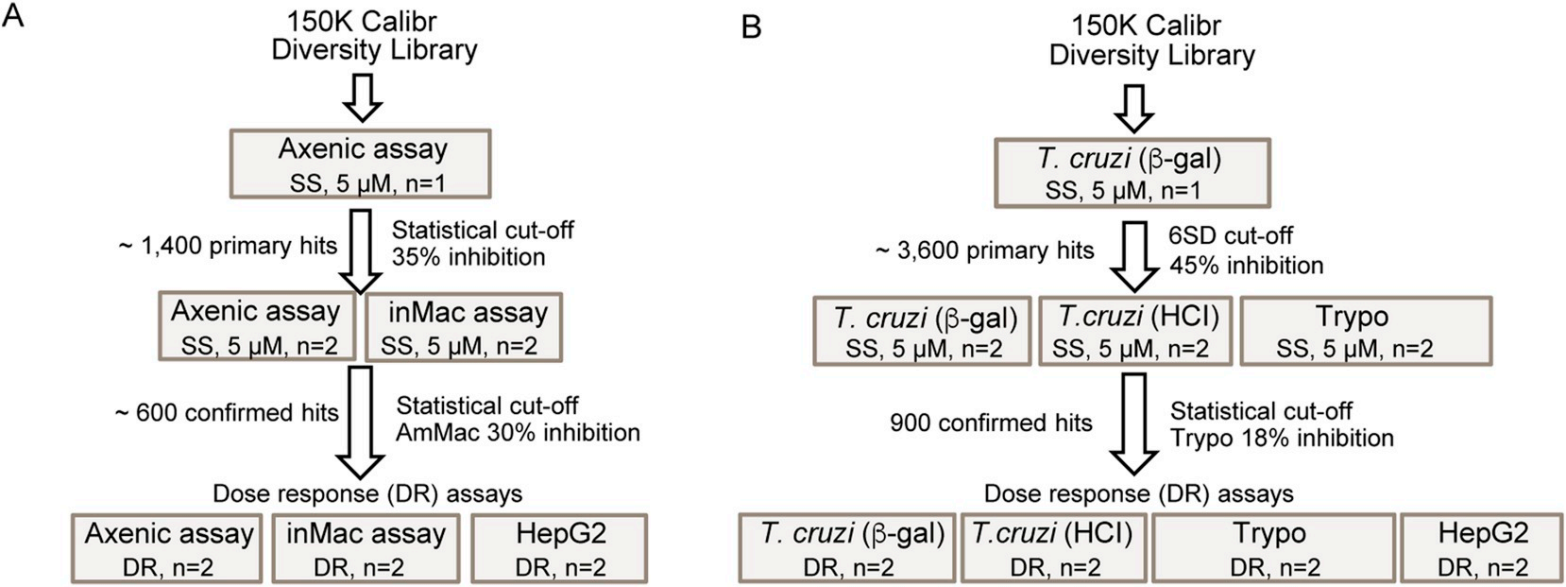
Progression cascade for the screening campaign against *L. donovani* (A) and *T. cruzi* (B). Compounds were either screened in a singular concentration (SS, single shot) of 5 μM or in dose-response (DR) format to generate half maximal inhibitory concentration (IC_50_). The number of technical replicates (n) are noted for each assay. Assay definitions: inMac = High-content imaging assay of *L. donovani* intramacrophage infection and proliferation; *T. cruzi* (β-gal) = *T. cruzi* trypomastigote β -galactosidase assay; *T. cruzi* (TcHCI) = High-content imaging assay of *T. cruzi* intracellular amastigote infection and proliferation; Trypo = *T. cruzi* trypomastigote viability assay (24, 48 or 72 h); HepG2 = 72 h mammalian cytotoxicity assay with HepG2 cell line.

**Fig. 2:**
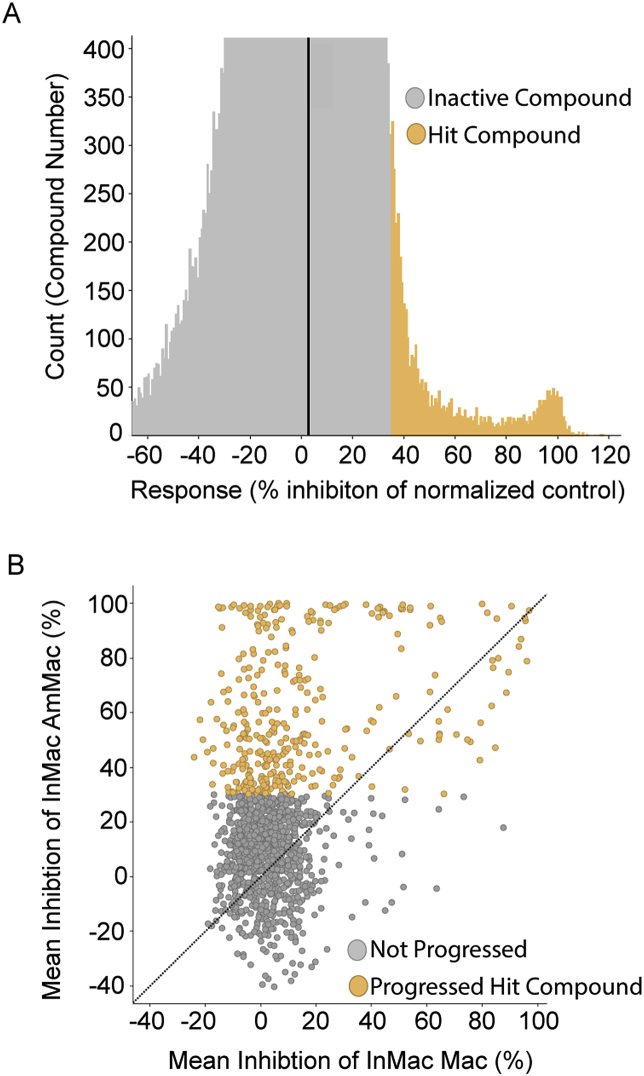
(A) Representation of compound distribution by % inhibition of *L. donovani* axenic amastigote growth in the primary screen. Inactive and active hit compounds are color-coded gray and orange, respectively. (B) Distribution of the hits selected from primary screen according to InMac assay results during single point confirmation phase. Amastigote-specific activity (y-axis; on-target effect) is plotted against the measured % inhibition of host macrophage cells (x-axis; off-target effect). Those compounds that were progressed (orange) or deprioritized for a lack of activity and/or selectivity (gray) are color-coded. (For interpretation of the references to color in this figure legend, the reader is referred to the Web version of this article.)

The screening progression cascade (Fig. 1A) devised for this workflow began with the determination of growth inhibition of *L. donovani* amastigotes in the axenic assay described in section 2.3.1. The average Z’ value for this HTS was 0.57 and a total number of 109 plates were assayed. Using a robust activity cut off of three standard deviations (3 × SD), 1392 hits were selected from the 150,000 compounds tested (hit rate near 1%) (Fig. 2A). These primary screen hits were reconfirmed against both the axenic and the more physiologically relevant InMac assays. Within the InMac assay, the robust 3 × SD activity cutoff for AmMac output (which correlated to 30% inhibition of parasite proliferation) produced 560 hits (Fig. 2B). Nitroaromatic compounds and PAINS (Pan-Assay Interference Compounds) were discarded to eliminate compounds with known toxicity liabilities (Baell and Holloway, 2010). Subsequent reconfirmatory dose-response assays against axenic amastigotes, InMac and HepG2 cell line (an additional representative mammalian cell line to further assess potential cytotoxicity) were performed in duplicate using resupplied powder stock of all available compounds (63 compounds in total). Application of potency (AmMac pIC50 > 5) and selectivity (Selectivity Index (SI) > 1) filters generated five chemotype families (Table 1; denoted as hit clusters A to E) and four singletons (Table 1) prioritized for additional biological profiling (Section 3.3).

**Table 1:** Hit compound structures and corresponding assay activities from the *L. donovani* and *T. cruzi* screening campaigns.

Activity^a^	Compound Id.	Structure	ID^b^ and priority^c^	***L. donovani***			***T. cruzi***		HepG2^e^ (pCC50)
				Axenic assay (pIC50)	InMac assay (pIC50)	InMac HS assay^d^ (pIC50)	High-content imaging assay (pIC50)	Trypomastigote assay (pIC50)	
Both parasites	TCOLFS068570		Cluster A (H)	5.93 ± 0.01	AmMac^f^ 6.3 ± 0.25 InfCel^g^ 6.27 ± 0.2 Mac^h^ <4.3	AmMac HS 6.17 ± 0.19 InfCel HS 6.09 ± 0.11 Mac HS 4.53 ± 0.28	AmCel 5.85 ± 0.11 InfCel 4.4 ± 0.25 H9c2^i^ < 4.3	24 h < 4.3 48 h < 4.3 72 h < 4.3	<4
	TCOLFS018919		Cluster B (H)	5.80 ± 0.15	AmMac 5.68 ± 0.30 InfCel 5.09 ± 0.65 Mac <4.3	AmMac HS 4.89 ± 0.32 InfCel HS 4.75 ± 0.30 Mac HS < 4.3	AmCel 5.71 ± 0.30 InfCel 5.38 ± 0.30 H9c2 <4.3	24 h < 4.3 48 h < 4.3 72 h 4.53 ± 0.27	<4
	TCOLFS026832		Cluster C (H)	6.53 ± 0.03	AmMac 6.47 ± 0.26 InfCel 6.32 ± 0.17 Mac 4.5 ± 0.31	AmMac HS 6.13 ± 0.05 InfCel HS 5.96 ± 0.09 Mac HS 4.62 ± 0.45	AmCel 7.98 ± 0.23 InfCel 7.64 ± 0.28 H9c2 <4.3	24 h 7.28 48 h 7.08 72 h 7.41 ± 0.19	<4
	TCOLFS026398		Cluster D (H)	- -	AmMac 5.09 ± 0.06 InfCel 5.03 ± 0.1 Mac <4.3	- -	AmCel 6.65 ± 0.2 InfCel 6.37 ± 0.2 H9c2 <4.3	24 h to −48 h to −72 h 6.06 ± 0.16	<4
	TCOLFS129266		Cluster E (L)	6.54 ± 0.01	AmMac 6.28 ± 0.33 InfCel 5.62 ± 0.78 Mac 4.90 ± 0.18	- -	AmCel 6.77 ± 0.01 InfCel 6.68 ± 0.03 H9c2 4.42 ± 0.02	24 h to −48 h to −72 h 6.24 ± 0.01	4.69 ± 0.01
	TCOLFS098882		Singleton (H)	5 ± 0.04	AmMac 5.82 ± 0.55 InfCel 5.2 ± 0.09 Mac 4.46 ± 0.22	- -	AmCel 5.37 ± 0.06 InfCel 5.17 ± 0.24 H9c2 5.61	24 h to −48 h to −72 h 5.66 ± 0.01	<4
	TCOLFS006487		Singleton (L)	5.23 ± 0.05	AmMac 5.64 ± 0.32 InfCel 5.38 ± 0.09 Mac 4.53 ± 0.25	- -	AmCel 5.89 ± 0.06 InfCel 5.79 ± 0.06 H9c2 5.28 ± 0.1	24 h to −48 h to −72 h 5.94 ± 0.02	4.33 ± 0.38
*L. donovani*	TCOLFS079469		Singleton (H)	6.38 ± 0.03	AmMac 6.25 ± 0.28 InfCel 4.62 ± 0.46 Mac <4.3	- -	AmCel <4.3 InfCel <4.3 H9c2 <4.3	24 h to −48 h to −72 h < 4.3	<4
	TCOLFS124301		Singleton (H)	5.51	AmMac 5.46 ± 0.04 InfCel 5.28 ± 0.08 Mac <4.3	- -	- -	- -	4.58 ± 0.82
*T. cruzi*	TCOLFS008553		Cluster F (H)	- -	AmMac <4.3 InfCel <4.3 Mac 4.42 ± 0.19	AmMac HS < 4.3 InfCel HS < 4.3 Mac HS 4.52 ± 0.27	AmCel 6.3 ± 0.11 InfCel 5.88 ± 0.11 H9c2 4.36 ± 0.08	24 h 5.66 ± 0.06 48 h 5.31 ± 0.08 72 h 5.44 ± 0.05	<4
	TCOLFS129639		Cluster G (L)	- -	AmMac 5.01 ± 0.17 InfCel 4.31 ± 0.01 Mac 4.74 ± 0.21	AmMac HS 4.78 ± 0.15 InfCel HS 4.52 ± 0.06 Mac HS 4.42 ± 0.16	AmCel 6.45 ± 0.11 InfCel 6.13 ± 0.27 H9c2 4.41 ± 0.12	24 h 4.8 48 h 5.27 72 h 5.84 ± 0.08	<4
	TCOLFS025993		Cluster H (M)	- -	AmMac 4.84 ± 0.21 InfCel 4.52 ± 0.24 Mac 4.36 ± 0.09	AmMac HS 4.64 ± 0.43 InfCel HS < 4.3 Mac HS 4.44 ± 0.28	AmCel 6.68 ± 0.2 InfCel 6.49 ± 0.19 H9c2 <4.3	24 h < 4.3 48 h < 4.3 72 h 5.59 ± 0.11	<4
	TCOLFS059386		Singleton (L)	- -	- -	- -	AmCel 5.9 ± 0.06 InfCel 5.82 ± 0.1 H9c2 <4.3	24 h to −48 h to −72 h 5.27 ± 0.01	<4
	TCOLFS089113		Singleton (L)	- -	AmMac <4.3 InfCel <4.3 Mac <4.3	AmMac HS 4.73 ± 0.01 InfCel HS 4.41 ± 0.16 Mac HS 4.89 ± 0.04	AmCel 6.3 ± 0.12 InfCel 6.03 ± 0.18 H9c2 <4.3	24 h < 4.3 48 h < 4.3 72 h 4.83 ± 0.77	<4
	TCOLFS135869		Cluster I (L)	- -	- -	- -	AmCel 6.85 ± 0.14 InfCel 6.48 ± 0.1 H9c2 <4.3	24 h to −48 h to −72 h 6.33 ± 0.06	<4
	TCOLFS112845		Singleton (M)	- -	- -	- -	AmCel 6.07 ± 0.04 InfCel 5.98 ± 0.07 H9c2 <4.3	24 h to −48 h to −72 h 5.27 ± 0.01	<4
	TCOLFS002713		Singleton (M)	- -	- -	- -	AmCel 6.27 ± 0.01 InfCel 5.97 ± 0.08 H9c2 <4.3	24 h to −48 h to −72 h 5.72 ± 0.01	<4
	TCOLFS050529		Singleton (L)	- -	- -	- -	AmCel 6.0 ± 0.01 InfCel 5.88 ± 0.08 H9c2 <4.3	24 h to −48 h to −72 h 4.42 ± 0.1	<4
	TCOLFS099080		Singleton (L)	- -	- -	- -	AmCel 6.35 ± 0.02 InfCel 5.87 ± 0.11 H9c2 <4.3	24 h to −48 h to −72 h 5.64 ± 0.01	4.73
	TCOLFS013976		Singleton (L)	- -	- -	- -	AmCel 6.13 ± 0.03 InfCel 5.76 ± 0.1 H9c2 <4.3	24 h to −48 h to −72 h 5.69 ± 0.01	<4
	TCOLFS148231		Singleton (M)	- -	- -	- -	AmCel 6.21 InfCel 5.87 ± 0.11 H9c2 4.85 ± 0.05	24 h to −48 h to −72 h 5.64	<4

- - not tested.

*Compounds determined to not be novel; novel compounds shown in Table 3.

a Compounds are classified per their antiparasitic activity (both parasites, *L. donovani*, or *T. cruzi*).

b Hits are categorized as either a hit cluster (2 or more related analogs; clusters A−I) or singletons (i.e., those hits with no related analogs).

c The hit priority level based on biological activity (H: high, meets desirable activity profile; M: medium, slow-acting compound or missing activity profiling; L: low, undesirable activity profile).

d HS denotes replacement of FBS with horse serum in the *L. donovani* inMac assay.

e Additional mammalian cytotoxicity was measured against HepG2 (hepatocellular carcinoma) cell line.

f AmCel: High-content imaging assay out measuring the number of amastigotes per cell.

g InfCel: High-content imaging assay out measuring the number of infected host cells per well.

h Mac: Macrophage host cell for *L. donovani* intracellular high-content imaging assay.

i H9c2: Embryonic cardiomyocyte host cell for *T. cruzi* intracellular high-content imaging assay.

Evaluation of assay data quality was assessed by calculating Z′ value and in all cases a Z’ value > 0.4 was achieved, which is viewed as an acceptable metric for generating dose-response data. Additionally, Amphotericin B and Miltefosine were included as controls to ensure reproducible assay sensitivity was maintained between assay plates and assay batches. The effect of these standard drugs were reproducible and generated comparable IC50 values in both the axenic and InMac assays. The high sensitivity of the intramacrophage high-content imaging assay permits a decreased assay incubation time and provides concomitant measurement of intracellular parasite numbers and host cell cytotoxicity.

### 3.2 *T. cruzi* HTS campaign

**Fig. 3:**
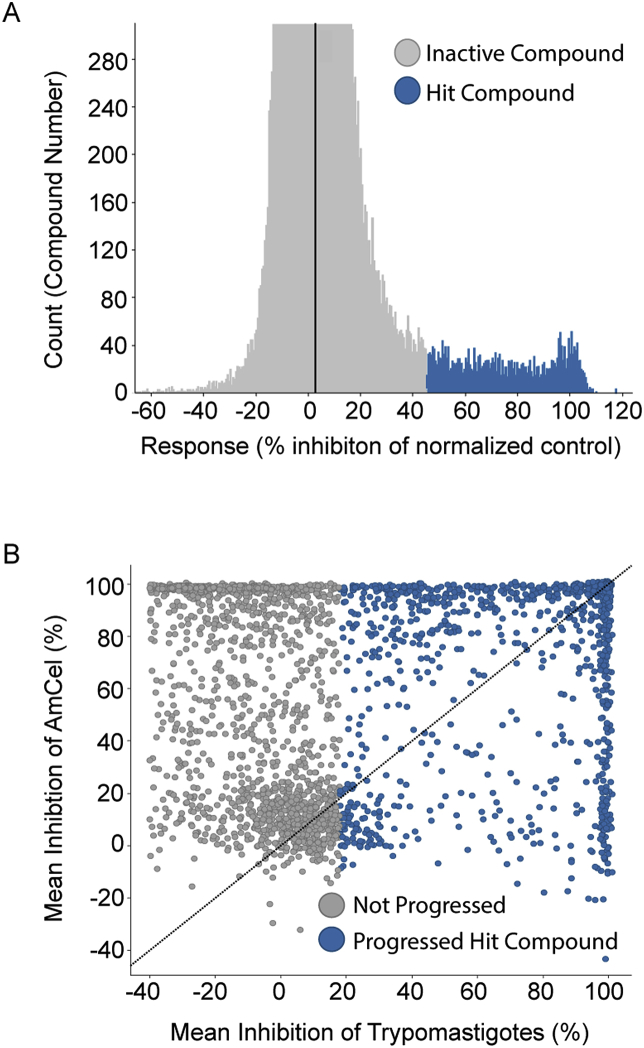
(A) Representation of compound distribution by % inhibition of *T. cruzi* growth measured in the β-galactosidase assay used for the primary screen. Inactive and active hit compounds are color-coded gray and blue, respectively. (B) Distribution of the hits selected from primary screen according to high-content imaging and 72 h trypomastigote assays results during single point confirmation phase. Those compounds that were progressed (blue) or deprioritized for a lack of activity against trypomastigote (≤20% inhibition) (gray) are color-coded. (For interpretation of the references to color in this figure legend, the reader is referred to the Web version of this article.)

The screening progression cascade of the *T. cruzi* HTS campaign (Fig. 1B) started with the determination of growth inhibition of *T. cruzi* parasites in the β-galactosidase assay described in section 2.4.1. This assay was preferred because of the high-throughput screening capacity in the BSL3 environment, allowing for testing of the entire 150,000 compound library. Importantly, active compounds in the β-galactosidase assay will target intracellular amastigotes in NIH-3T3 cells, though to a smaller extent they may also target free swimming trypomastigotes, the invasive form, present in the culture medium, and/or inhibit essential host-parasite interactions required for successful parasite invasion (Peña et al., 2015). The average Z’ value for HTS was 0.85 and a total number of 109 plates were assayed. Following screen analysis, 3598 compounds were selected as primary hits showing inhibition greater than 45% (cut-off at six standard deviations (6 × SD) (Fig. 3A)

These hit compounds were tested in duplicate for confirmation in the β-galactosidase, a high-content imaging assay of intracellular infection and the trypomastigote assay, described in section 2.4. Hit advancement criteria were defined by activity confirmation in both the β-galactosidase and high-content imaging assay, and a statistical cut-off of 18% inhibition in trypomastigote assay at 72 h incubation time. This resulted in the selection of 1337 compounds (Fig. 3B). Due to the high number of hit compounds, structural similarity clustering (Tanimoto cut-off of 80%) was used to identify highly related chemical scaffolds. Within each cluster, a maximum of three compounds were selected and prioritized according to the following calculated properties: lipophilicity, solubility, efflux pump substrate and volume of distribution. As with the *L. donovani* HTS campaign, nitroaromatic compounds and PAINS (Pan-Assay Interference Compounds) were discarded, reducing the total number of hits for progression to dose response assay formats to 900 compounds (Baell and Holloway, 2010). Of those 900, compounds were resupplied as powder stocks for reconfirmation testing in dose-response format amongst a quartet of assays: β-galactosidase activity, high-content imaging of intracellular infection, trypomastigote activity and HepG2 cytotoxicity. In total, 76 compounds were resupplied for dose-response reconfirmation. Nine chemotype families and ten singletons (Table 1) were selected based on efficacy output (AmCel pIC50 > 6 in at least one duplicate; a conservative approach to minimize the loss of compounds from false-negative activity), with a selectivity index greater than 1, and confirmatory activity in the trypomastigote assay.

Consistent with the *L. donovani* HTS campaign, evaluation of *T. cruzi* assay data quality was measured using Z’ and in all cases values were greater than 0.4. Nifurtimox, benznidazole and posaconazole were included as control compounds in each run to validate the quality of the assay. The control compounds displayed comparable activity in both the β-galactosidase and high-content imaging assay of intracellular infection despite the different incubation times. We speculate that the comparable activity between assays is attributable to greater accuracy of the high-content imaging assay of intracellular infection, which can detect compound efficacy in a shorter duration than the whole well, fluorescence-based readout used for the β-galactosidase assay.

Aligned with the objective of finding novel starting points to treat Chagas disease, nitroaromatic compounds were deprioritized at the confirmation phase. It is well known that nitroaromatic compounds such as benznidazole and nifurtimox cause severe adverse effects that may lead to treatment discontinuation. In addition, both benznidazole and nifurtimox are prodrugs that share their activation process through the mitochondrial nitroreductase of *T. cruzi*. This provides potential for cross-resistance and underscores the importance of identifying new drugs that target different biochemical pathways (Francisco et al., 2015). Furthermore, this compound class is already well represented in the drug discovery pipeline for Chagas disease. Considering compounds such as fexinidazole, currently undergoing clinical trials, and despite the advances, other novel chemical entities are still needed (Moraes et al., 2014).

One of the major challenges of Chagas disease drug discovery is the poor understanding on the key drivers to guide translation between *in vitro* and *in vivo* models and clinical outcomes. An exemplar is the unfortunate failure of posaconazole in clinical trials. Different causes have been attributed as reasons for this failure, such as incorrect dosing regimen (Urbina, 2017) or differential susceptibility of *T. cruzi* DTU strains (Francisco et al., 2015; Moraes et al., 2014). However, unlike benznidazole, posaconazole is trypanostatic and this is viewed as an unfavourable attribute for anti-Chagas drugs. In an attempt to differentiate and prioritize these novel chemotypes, trypomastigote cidality was assessed to determine whether these compounds profiled more closely with trypanostatic CYP51 inhibitors (e.g. posaconazole) or the cidal nitroaromatic compounds (e.g. nifurtimox and benznidazole). It has been demonstrated that CYP51 inhibitors have maximal activity against replicating parasites whereas minimal activity is observed against trypomastigote stage parasites (>3-log decrease in potency) (MacLean et al., 2018). This is an alternative approach to the epimastigote-based assay described by Sykes et al. to identify CYP51 inhibitors (Sykes and Avery, 2018).

### 3.3 Biological profiling of selected hits

**Fig. 4:**
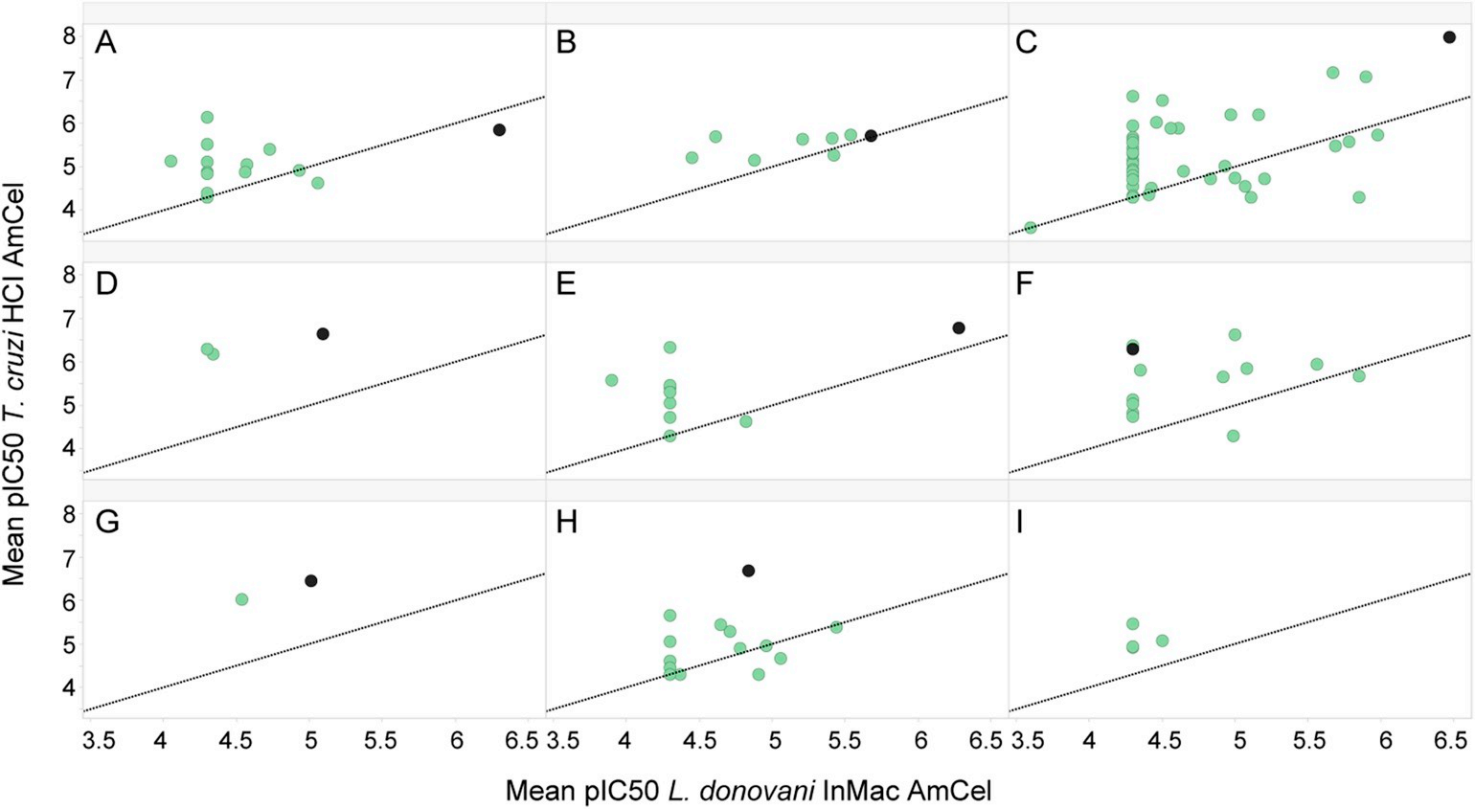
Activity potency distribution of the hit clusters (A−I; See Table 1) against amastigotes from *L. donovani* and *T. cruzi*. The activity of the initial hit compound (black) is shown against two key intracellular assays from each representative kinetoplastid: *L. donovani* InMac AmMac (the number of amastigotes per macrophage (AmMac) in the *L. donovani* intramacrophage assay (InMac)) and *T. cruzi* HCI AmCel (the number of amastigotes per cell) outputs. Resupplied analogs of the initial hit are represented in green. These data were only generated for compounds in which powder resupply was made available for dose-response reconfirmation testing. (For interpretation of the references to color in this figure legend, the reader is referred to the Web version of this article.)

The selected hits available for resupply were profiled in up to three general assay formats: high-content imaging assays of intracellular amastigote infection to reconfirm activity, *T. cruzi* trypomastigote assay at 24, 48 and 72 h and *L. donovani* InMac supplemented with horse serum (HS) (Table 1). To complement this activity profiling of the resupplied hit compounds, all available hit analogs were also profiled against the standard high-content imaging (HCI) assays of intracellular amastigote infection and *T. cruzi* trypomastigotes at 72 h (Table 2). All resupplied hit compounds reconfirmed activity against at least one of the two parasite species and activities ranged from sub-micromolar (pIC50 > 6) for the most potent hits to low, single-digit micromolar (5 > pIC50 > 6) IC50 values for the least potent chemotypes (Table 1). The activity profiling lead to binning the hits into three categories: (1) active against both parasites (pIC50 > 5 for intracellular Ld or Tc HCI assays), (2) *L. donovani*-specific hits (pIC50 > 5 for Ld HCI, pIC50 < 5 for Tc HCI), or (3) *T. cruzi*-specific hits (pIC50 > 5 for Tc HCI, pIC50 < 5 for Ld HCI). Cluster C was notable simply for its exceptional, low nanomolar activity against intracellular *T. cruzi*. As expected, extended testing of chemically related analogs showed a diverse range of activity, spanning from inactive analogs to those with comparable or even superior activity of the original hit compound (Table 2) (Fig. 4).

**Table 2:** Assay activity summary of the nine hit clusters identified from the *L. donovani* and *T. cruzi* screen campaign. “Active compounds” are those with AmMac or AmCel pIC_50_ > 5 and only selective compounds were considered (Mac or H9c2 pIC_50_ < 5).

Cluster ID	Compounds tested	*L. donovani*		*T. cruzi*		
		Number of active compounds	InMac assay pIC_50_ range	Number of active compounds	Imaging assay pIC_50_ range	Trypomastigotes assay 72 h pIC_50_ range
A	15	2	AmMac 5.06–6.30 InfCel 4.91–6.27 Mac <4.3	6	AmCel 5.05–5.85 InfCel <4.3–4.91 H9c2 < 4.3–4.77	<4.3–4.88
B	8	5	AmMac 5.21–5.68 InfCel 4.91–5.19 Mac <4.3–4.6	8	AmCel 5.16–5.73 InfCel 4.74–5.38 H9c2 < 4.3–4.52	<4.3–5.13
C	58	10	AmMac 5.0–6.47 InfCel 4.62–6.32 Mac <4.3–4.87	23	AmCel 5.02–7.98 InfCel <4.3–7.64 H9c2 < 4.3–4.96	<4.3–7.41
D	3	1	AmMac 5.09 InfCel 5.03 Mac <4.3	3	AmCel 6.18–6.65 InfCel 5.81–6.37 H9c2 < 4.3	5.54–6.06
E	13	1	AmMac 6.28 InfCel 5.62 Mac 4.9	7	AmCel 5.06–6.77 InfCel <4.3–6.68 H9c2 < 4.3–4.42	<4.3–6.24
F	13	4	AmMac 5.0–5.85 InfCel <4.3–5.15 Mac <4.3–4.86	10	AmCel 5.04–6.63 InfCel 4.43–6.24 H9c2 < 4.3–4.51	4.35–5.92
G	2	1	AmMac 5.01 InfCel 4.31 Mac 4.74	2	AmCel 6.02–6.45 InfCel 5.82–6.13 H9c2 < 4.3–4.41	5.41–5.84
H	19	1	AmMac 5.44 InfCel 5.39 Mac 4.51	6	AmCel 5.05–6.68 InfCel 4.97–6.49 H9c2 < 4.3–4.4	<4.3–5.59
I	5	0	- -	3	AmCel 5.07–6.85 InfCel <4.3–6.48 H9c2 < 4.3–4.51	<4.3–6.33

In the case of *L. donovani* hits, care was taken to directly assess compound activity against intracellular replication of amastigotes. This was achieved by replacing FBS with HS, and the change in serum prevents extracellular parasites from successfully re-establishing a new intramacrophage infection. This alteration to the *in vitro* assay conditions has been previously shown to have a positive correlation with *in vivo* efficacy (Tegazzini et al., 2016). Unfortunately, compound availability restrictions limited profiling for all chemotypes; however, comparative activity profiling between FBS-supplemented and HS-supplemented media showed comparable activity for all series tested.

Finally, the *T. cruzi* trypomastigote assay was performed using three different incubation times to obtain information about the speed of action of the compounds together with cidal/static assessment. Compounds active at any time point in the trypomastigote assay are considered cidal, and those active in short incubation times (ideally maximal activity in the 24-h time point) would be prioritized as fast-kill compounds. Examination of the dual-parasite active compounds revealed Clusters A to be inactive at all time points and therefore designated as a very low priority for Chagas drug development. Cluster B, an inherently weak compound hit, only demonstrated detectable activity at the final 72-h time point, which suggests it may be slow-acting, whereas Cluster C confirmed a ‘fast-kill’ compound profile from the dual-parasite active list. Amongst the *T. cruzi*-specific hits, Cluster H demonstrated a slow onset of action (i.e. no detectable activity until the 72-h time point) whereas the other two hit clusters were categorized as ‘fast-kill’ compounds. Kill kinetics were not assessed for hit singletons and only activity at the 72-h time point was measured. Singletons TCOLFS089113 and TCOLFS050529 were the only hit compounds that failed to show significant activity against trypomastigotes and therefore merit deprioritization relative to the other active hit compounds because of the lack of cidality.

Further structural comparison of these reconfirmed compound hits to previously disclosed chemotypes (Peña et al., 2015) revealed high similarity to known kinetoplastid-active compounds (Table 3). Although the rediscovery of known compounds eliminates their novelty, their discovery also validates the success of our workflow. Further review of historical screen data at Calibr for *Plasmodium falciparum* (human malaria) erythrocytic stages (unpublished data), *Cryptosporidium parvum* intracellular asexual stages (Love et al., 2017), *Wolbachia*-infected *Drosophila* (Bakowski et al., 2019), and *Mycobacterium tuberculosis* broth and intramacrophage assays (unpublished data) did not identify these chemical scaffolds as hits. These observations were further validated using a cheminformatic approach. Reaxys, a web-based database of publicly disclosed compounds (http://www.reaxys.com), was used to perform similarity and substructure searches of the compound hits listed in Table 1. Of the twenty-one compounds searched, nine compounds (see Table 3) were discovered to have had prior disclosures for kinetoplastid activity. A tenth compound, the 1,5-benzodiazepine substructure representative of Cluster A (TCOLFS068570) has previously been reported as an allosteric inhibitor of NS5B polymerase in hepatitis C virus (McGowan et al., 2009; Vandyck et al., 2009). Yet, no reports for kinetoplastid drug discovery were identified. All remaining twelve compounds had no associated publication/public disclosure, supporting the specific and selective nature of these compounds for kinetoplastid drug discovery.

**Table 3:** Hit compounds in which anti-kinetoplastid activity was previously reported.

Compound ID	Cluster Id.	Analog	Reference
TCOLFS129266	E	TCMDC-143197	Peña et al. (2015)
TCOLFS006487	Singleton	TCMDC-143247	
		TCMDC-143097	
TCOLFS059386	Singleton	TCMDC-143193	
		TCMDC-143194	
TCOLFS089113	Singleton	TCMDC-143426	
TCOLFS135869	I	TCMDC-143248	
TCOLFS050529	Singleton	TCMDC-143465	
TCOLFS099080	Singleton	TCMDC-143109	
TCOLFS013976	Singleton	TCMDC-143511	
TCOLFS129639	G	GNF6702	(Khare et al., 2016)

Based on the novel activity against *L. donovani* and/or *T. cruzi* without prior disclosure and the activity criterion we established for hit prioritization, the finalized list of chemical starting points numbered twelve chemotypes in total (6 singletons and 6 hit clusters; Clusters A−D, F, H and singletons TCOLFS098882, TCOLFS079469, TCOLFS124301, TCOLFS112845, TCOLFS002713, and TCOLFS148231). Interestingly, five of these prioritized chemotypes (hit clusters B−D, singletons TCOLFS098882 and TCOLFS006487) demonstrate activity against the amastigote stage of both kinetoplastid species. With the exception of Cluster A, all *T. cruzi*-active compounds demonstrated trypomastigote cidality, which we deemed an essential property for a chemical starting point for Chagas drug discovery. Presumably, the novelty of these compounds may also result in the identification of equally novel drug targets. Thus, we also view these compounds as powerful chemical tools to probe parasite biology and target identification.

The joint discovery of novel anti-kinetoplastid compounds between our institutes (TCOLF and Calibr) is an exemplar of the scientific advances that can be accomplished in the context of open innovation, and disclosure of these screen results, including compound structures, are meant to encourage continued drug development for two, important, neglected tropical diseases. As next steps, medicinal chemistry is required for resynthesis of the highest priority compounds for reconfirmation of biological activity and to enable structural confirmation of hit compounds. Resynthesis will provide sufficient powder stock to submit compounds to *in vitro* ADME assays, particularly liver microsomal stability data (mouse, rate and human), kinetic solubility, Caco-2 permeability assay, and human CYP inhibition panel (CYP1A, CYP2B6, CYP2C8, CYP2C9, CYP2C19, CYP2D6 and CYP3A4). These assays would be useful to further characterize the drug-like properties of these compound series and further prioritize candidates for hit-to-lead chemistry.

## Conflicts of interest

The authors declare no conflict of interest.
